# Transthyretin Cardiac Amyloidosis in Women: Underdiagnosis, Sex-Specific Phenotypic Expression and Therapeutic Response

**DOI:** 10.3390/jcm15156033

**Published:** 2026-08-03

**Authors:** Federico Barocelli, Eleonora Canu, Giovanni Tassoni, Angelo Mastrangelo, Nicolò Pasini, Antonio Crocamo, Filippo Luca Gurgoglione, Laura Torlai Triglia, Francesca Russo, Angela Guidorossi, Maria Francesca Notarangelo, Gian Luca Gonzi, Nicola Gaibazzi, Giampaolo Niccoli

**Affiliations:** Cardiology Division, Parma University Hospital, 43126 Parma, Italy; eleonora.canu@unipr.it (E.C.); giovanni.tassoni@unipr.it (G.T.); angelo.mastrangelo@unipr.it (A.M.); nicolo.pasini@unipr.it (N.P.); acrocamo@ao.pr.it (A.C.); filippolucagurgoglione@gmail.com (F.L.G.); laura.torlait@gmail.com (L.T.T.); russofr@ao.pr.it (F.R.); aguidorossi@ao.pr.it (A.G.); notarangelof@ao.pr.it (M.F.N.); ggonzi@ao.pr.it (G.L.G.); ngaibazzi@gmail.com (N.G.); giampaolo.niccoli@unipr.it (G.N.)

**Keywords:** transthyretin cardiac amyloidosis, sex differences, cardiac amyloidosis, hormonal and genetic factors, diagnostic accuracy

## Abstract

Transthyretin cardiac amyloidosis (ATTR-CA) is an increasingly recognized cause of cardiac dysfunction in adults, resulting from extracellular deposition of misfolded transthyretin fibrils and progressive myocardial impairment. Clinical expression and diagnostic yield differ substantially between sexes, contributing to systematic underdiagnosis in women, who often present with subtler myocardial remodeling, heart failure with preserved ejection fraction (HFpEF)–dominant phenotypes, and nonspecific systemic manifestations that fall below conventional diagnostic thresholds, particularly in early disease stages. Female patients, particularly those with ATTRwt, tend to present at older ages and more frequently show HFpEF-dominant phenotypes and nonspecific extracardiac manifestations. Carpal tunnel syndrome (CTS) is an important extracardiac red flag for ATTR-CM, but its interpretation in women requires caution because of the high background prevalence of idiopathic CTS in the general population. Evidence also suggests sex-related differences in diastolic function, right ventricular involvement, and overall progression. Despite these biological and phenotypic distinctions, women are markedly underrepresented in trials of disease-modifying therapies, limiting conclusions about sex-specific treatment effects and leaving uncertainty about whether current pharmacologic interventions provide comparable benefit. Hormonal influences, genetic background, and age-related mechanisms, comorbidities, and diagnostic pathways may contribute to the distinctive female phenotype, but underlying mechanisms remain insufficiently defined. This narrative review examines sex-associated differences in ATTR-CA, focusing on mechanisms of underdiagnosis, principal clinical and imaging features, and implications for therapeutic response, with the goal of improving diagnostic accuracy, guiding individualized management, and ultimately enhancing outcomes for women and all affected patients worldwide in clinical practice.

## 1. Introduction

Systemic amyloidosis refers to a heterogeneous group of diseases caused by the deposition of amyloid fibrils in the extracellular space of various organs. These fibrils can be composed of more than 30 protein precursors [1]. Nevertheless, the main causes of cardiac amyloidosis are five misfolded proteins: immunoglobulin light chain (light chain amyloidosis [AL]), immunoglobulin heavy chain (AH), transthyretin (ATTR), serum amyloid A (AA), and apolipoprotein A-I (AApoA1) [2]. These precursors are capable of misfolding and self-assembling into a highly ordered, abnormal, cross-β-sheet conformation, forming insoluble fibrillar material with a relatively stable core structure resistant to proteolysis [1]. Histologically, amyloid deposits are identifiable by their characteristic apple-green birefringence when stained with Congo red dye and observed under polarized light.

### 1.1. ATTR-CA: Pathophysiology, Genotype and Phenotype

Transthyretin (TTR) is a tetrameric protein with β-sheet–rich monomers synthesized predominantly in the liver and, to a lesser extent, in the choroid plexus and retinal epithelium. In physiological conditions, it functions as a transporter of thyroxine and retinol–vitamin A–binding protein complexes [3]. TTR monomers may undergo unfolding, subsequent misfolding and loss of tetrameric integrity, ultimately aggregating to form amyloid fibrils. Misfolded TTR commonly accumulates within the interstitial spaces of the myocardium, leading to cardiac amyloidosis. In this situation the amyloid fibrils disrupt the structure, integrity, and function of the organ causing interruption and distortion of myocardial contractile elements [4]. Following protein deposition, the myocardium becomes hypertrophied, with a concomitant reduction in ventricular compliance, progressing to restrictive cardiomyopathy. The diastolic dysfunction arising from this pathological aggregation results in worsening heart failure, the development of new conduction abnormalities, and arrhythmias [3]. Two types of cardiac amyloidosis are recognized. Wild-type TTR (ATTRwt)-cardiac amyloidosis, due to amyloid deposition derived from non-mutated TTR, which leads to amyloid deposition in cardiac tissues. By contrast variant TTR amyloidosis (ATTRv) is secondary to point mutations within the nucleotide sequences responsible for the transcription and translation of TTR, transmitted in an autosomal dominant pattern and due to one of the more than 130 mutations in the TTR gene on chromosome 18) [2,5]. In this latter form of cardiac amyloidosis, TTR mutations alter dissociation kinetics, thereby promoting amyloid formation. According to the THAOS (Transthyretin Amyloidosis Outcomes Survey) registry [6], the most frequent mutations involved in ATTRv are p.Val50Met (Val30Met) 28%, p.Ile88Leu (Ile68Leu) 8%, p.Leu131Met (Leu111Met) 5%, p.Val40Ile (Val20Ile) 4%, p.Gly67Ala (Gly47Ala) 4%, p.Val142Ile (Val122Ile) 1%, other (24%). From the most recent data [7], the p.Val50Met genotype remains the most prevalent among patients enrolled in the THAOS study (48.0%), followed by ATTRwt (25.2%) and the p.Val142Ile mutation (6.0%). Among symptomatic patients, the p.Val50Met genotype was most frequently observed in Europe (54.2%), South America (79.5%), and Japan (75.4%), whereas wild-type disease was widely observed in North America (59.5%). Non-p.Val50Met variants were more common (sometimes even more prevalent than the wild-type form) in individual countries such as Mexico, Bulgaria, Denmark, Israel, Italy, Romania, Turkey, Malaysia, and Taiwan [7]. In ATTRv there is considerable heterogeneity in disease presentation. Phenotypes can be predominantly neuropathic, predominantly cardiac or mixed [6]. Similarly to ATTR-CA wt, the p.Val142Ile mutation leads to a clinically significant amyloid deposition predominantly involving the heart. Compared with ATTR-CA wt, patients with ATTRv due to the p.Val142Ile mutation are older, exhibit less severe symptoms, and show greater ventricular hypertrophy, more pronounced atrial dilation, and lower left ventricular ejection fraction. Conversely, TTR gene mutations endemic to Europe and Asia may cause cardiac involvement associated with familial amyloid polyneuropathy (FAP), a syndrome characterized by articular, peripheral nervous system, and muscular involvement. In transthyretin cardiac amyloidosis (ATTR-CA) the cardiac phenotype patterns vary considerably. Overall, this condition coexists in approximately 12% of patients with severe aortic stenosis referred for valve replacement and is frequently an unrecognized cause of HFpEF (it is identified in 6% of older community-dwelling patients with increased wall thickness ≥ 12 mm) [8].

### 1.2. Rationale for a Sex-Specific Perspective

Male predominance is well recognized in clinically diagnosed ATTR-CA, particularly in ATTRwt. In ATTRv, however, sex-related differences should not be interpreted as reflecting a higher prevalence of pathogenic TTR variant carriage in men. Rather, available registry data suggest that male predominance is mainly observed among symptomatic patients and in those with predominantly cardiac phenotypes, supporting sex-related differences in penetrance, cardiac phenotypic expression, and severity of myocardial involvement. Realistically, female patients remain underdiagnosed. In a recent study investigating sex-related differences [9], female prevalence was found to be higher in ATTRv compared with ATTRwt, and women were, on average, older at the time of clinical presentation. Moreover, no marked differences in clinical phenotype were observed, nor was there any significant difference in mortality between males and females in the overall population. From an echocardiographic standpoint, after indexing for body surface area (BSA), structural and functional characteristics were comparable between sexes, although women exhibited a slightly worse phenotype at the time of diagnosis. Current literature does not clearly define sex-related differences in disease progression, including disease severity at clinical presentation and survival. Indeed, previous retrospective and prospective studies have reported conflicting results regarding sex differences in phenotypic expression (particularly echocardiographic presentation) as well as in disease severity and long-term prognosis [9]. Considering this, it may be necessary to take a gender-focused view of the topic.

### 1.3. Scope and Objectives of the Narrative Review

The aim of this narrative review is to delineate sex-associated differences in ATTR-CA, with a particular focus on the female phenotype. We highlight mechanisms contributing to underdiagnosis, describe key clinical and imaging characteristics, and discuss potential implications for therapeutic response. This review seeks to improve diagnostic accuracy, inform individualized management strategies, and ultimately enhance clinical outcomes for women and, by extension, all patients affected by ATTR-CA.

## 2. Epidemiology of ATTR-CA: Sex Distribution and Diagnostic Delay

### 2.1. Sex-Specific Prevalence Across Registries

Our knowledge of the epidemiology of cardiac amyloidosis is based mostly on real-world studies and on registries of diagnosed patients. In a meta-analysis of screening studies [10] (involving 31 articles: 6 with retrospective design, 21 with prospective design and 4 were forensic series) 10, the diagnosis of ATTR-CA was suspected based on CMR, bone scintigraphy, through multiparametric algorithms or histology. It was seen that ATTR-CA is common in several clinical settings (HFpEF, HFmrEF, HFrEF, conduction disorders requiring pacemaker implantation, hypertrophic cardiomyopathy and severe aortic stenosis), and its prevalence increases with age. Men represented a high proportion of patients with CA, suggesting that female sex may indicate a protective effect. Nonetheless, women represented 27% of patients with HFpEF, 36% of those with CTS surgery and 33% of patients with severe AS [10]. In the THAOS observational study [7], as of 2023, a male predominance was observed in all subgroups among symptomatic patients. Specifically, 93.3% in ATTRwt-CA, 64.6% in p.Val50Met late-onset and 65% in non p.Val50Met mutated. In contrast, among patients with early-onset p.Val50Met ATTR-CA (more “neuropathic” variant), 52.2% were male and 47.8% were female. In asymptomatic carriers, the highest percentage was female. This latter consideration may highlight a lower disease penetrance in women, especially in the cardiac involvement of TTR amyloidosis. It is worth noting that male predominance at diagnosis is less pronounced in older individuals. In a recent meta-analysis on gender prevalence in ATTRwt-CA, the higher proportion of males at diagnosis was more evident among patients aged < 80 years compared with those aged ≥ 80 years (92.7% vs. 69.5%, respectively) [11].

### 2.2. Age at Diagnosis and Diagnostic Latency in Women

Typically, women are older than men at the time of diagnosis. This likely occurs due to a potentially slower disease progression, underdiagnosis of the condition, and the protective effect exerted by estrogens throughout life [12]. Evidence of the underdiagnosis of cardiac amyloidosis in females also derives from post-mortem findings, in which it was observed that patients who received a post-mortem diagnosis were more likely to be older and female compared with those diagnosed during life [13].

The main reasons of diagnostic delay in women are various:

- The disease is believed to predominantly affect elderly men.
- From an echocardiographic standpoint, women exhibit lower interventricular septal and posterior wall thickness compared with their male counterparts, as clearly demonstrated in the study by Aimo et al. [14]. Indeed, diagnostic suspicion is usually raised in the presence of a left ventricular wall thickness ≥ 12 mm associated with at least one “red flag”. However, since normal wall thickness values are lower in women, this approach may lead to delayed diagnosis. Considering that women typically display a less hypertrophic phenotype in amyloid cardiomyopathy, morphological parameters on cardiac ultrasound should be indexed to BSA. A simpler alternative would be to calculate a cut-off for women based on the mean height of men and women in Europe (1.77 m and 1.65 m, respectively); given 12 mm the cut-off for men, the corresponding cut-off for women would be 11 mm. By indexing, women have a similar if not greater parietal thickness than men [14].
- In recent years, myocardial SPECT has become the primary diagnostic tool for ATTRwt. In the work of Takashio et al. [15], reduced myocardial uptake of technetium-99m pyrophosphate (99mTc-PYP) was observed in women. This obviously slows the diagnostic process and makes it more difficult. In fact, in the cited study, the mean heart-to-contralateral ratio obtained using 99mTc-PYP was significantly lower in women than in men (1.64 vs. 1.89).

Evidence on sex-specific ECG and CMR findings remains limited and heterogeneous. Earlier studies did not provide robust sex-stratified ECG or CMR data, whereas more recent reports suggest possible sex-related differences in CMR-derived amyloid burden and conduction abnormalities [16].

### 2.3. Implications for Disease Recognition

Clearly, the recognition of red flags remains fundamental in raising diagnostic suspicion. In women, a greater degree of disability due to polyneuropathy has been observed (increased proportion of sensory abnormalities) whereas in men there is a greater autonomic impairment, as seen in THAOS registry [7]. Similarly, CTS is common in women in the general population, but its diagnostic specificity as a red flag for ATTR-CM may be lower in women than in older men, potentially contributing to under-recognition [12].

The relationship between myocardial ATTR amyloid deposition, severe aortic stenosis, and a possible female predominance remains controversial, largely because published studies have yielded conflicting results and are frequently limited by small sample sizes. In the study by Takashio et al. [15], women exhibited a higher prevalence of moderate-to-severe aortic stenosis and were significantly older at the time of diagnosis than women without aortic stenosis. In the exploratory study by Furukawa et al. [17], multivariate analysis demonstrated that increased myocardial ATTRwt amyloid deposition was an independent determinant of severe aortic stenosis in the overall cohort. However, it is important noting that the study enrolled only 39 patients (4 women), of whom only 5 had severe aortic stenosis (1 woman). Indeed, the prevalence of both ATTR-CA and AS increases with age, and several studies have reported ATTR-CA in patients with severe aortic stenosis [17]. In the study by Scully, Fontana, et al. [18], 101 patients undergoing the TAVI pathway were enrolled and underwent DPD scintigraphy. ATTR cardiac amyloidosis (ATTR-CA) was diagnosed in 14 patients (13.9%). In this cohort, the prevalence of amyloidosis was similar between men and women (16% in men vs. 12% in women), consistent with previous observations in patients with heart failure with preserved ejection fraction (HFpEF). These findings further confirmed the earlier study by Castaño et al. [19], in which 16% of patients undergoing TAVR were found to have occult cardiac amyloidosis. Nevertheless, patients with severe aortic stenosis and ATTR-CA were predominantly male (92%). In a meta-analysis by Riley et al. [20]. comprising 83 patients with concomitant CA and AS (30% women), TAVI was associated with significantly lower all-cause mortality compared with conservative medical therapy (OR 0.24; 95% CI 0.08–0.73), supporting the benefit of valve intervention regardless of sex, although sex-stratified outcomes were not reported. These findings underscore that while AS may represent an important pathway to ATTR-CM diagnosis in women—particularly in elderly patients referred for TAVR—the strength of this association is largely derived from specific screening cohorts rather than from population-based studies, and further sex-stratified data are needed to clarify whether AS is disproportionately prevalent in female ATTR-CM.

Moreover, women more often present with HFpEF at the time of diagnosis and, compared with men, exhibit a higher NYHA functional class. Conversely, there does not appear to be a difference in the occurrence of atrial fibrillation. Still in the Japanese study by Takashio et al. [15], from the laboratory point of view, no significant differences were observed between the two sexes in median hs-cTnT levels, whereas higher BNP levels were detected in women (394 vs. 236 pg/mL). Additionally, hemoglobin levels and eGFR were lower at the time of diagnosis in female patients. However, in the THAOS cohort, no significant gender differences were found in NT-proBNP levels in either ATTRwt or ATTRv forms [7]. These apparently divergent findings should be interpreted cautiously, because natriuretic peptide concentrations are influenced not only by myocardial amyloid burden but also by several other factor such age, renal function, atrial fibrillation, hemodynamic loading conditions, and body size.

## 3. Potential Biological Determinants of Sex Differences in ATTR-CA

### 3.1. Hormonal Influences

Clinical and registry data have consistently reported sex-related differences in transthyretin cardiac amyloidosis (ATTR-CA), with men representing the majority of diagnosed cases and women often presenting at an older age; these observations have prompted interest in endocrine modifiers of disease expression, although causal mechanisms in humans remain incompletely defined [12,21]. A clinically relevant observation is that, in an observational cohort, women with greater myocardial involvement were more frequently postmenopausal, whereas no analogous age-related association was reported in men; this association is hypothesis-generating and does not establish a cause–effect relationship between estrogen status and amyloid deposition [22].

Human population data also demonstrate sex differences in circulating transthyretin: in a UK Biobank analysis including 35,206 participants, transthyretin levels were lower in females than in males, and the authors postulated that sex hormones could contribute to sex differences in hepatic transthyretin production, citing animal-model evidence of hormone-regulated transthyretin expression [23]. Taken together, these clinical and population findings have been used to hypothesize that sex hormones may modify ATTR-CA expression, but they do not demonstrate direct hormone-mediated effects on myocardial amyloid deposition in humans [12].

Separately from amyloid-specific mechanisms, estrogen signaling has documented effects on myocardial remodeling; in experimental work, 17β-estradiol inhibited angiotensin II-induced cardiac hypertrophy and interstitial fibrosis via estrogen receptor-β signaling [20,21,22,23]. Experimental studies in mice show that sex hormones can upregulate transthyretin production: both 17β-estradiol and 5α-dihydrotestosterone increased hepatic transthyretin gene transcription and circulating transthyretin concentrations [24,25]. In separate rodent experiments, 5α-dihydrotestosterone also increased transthyretin levels in the choroid plexus [26].

ATTRwt, historically referred to as senile systemic amyloidosis, has traditionally been reported as predominantly affecting older men. Nevertheless, recent diagnostic and autopsy data indicate that the observed male predominance may partly reflect ascertainment bias, while more severe myocardial infiltration in men may still suggest a role for biological sex-related modifiers. In a transgenic, seeded model of human transthyretin amyloidosis (TTRS52P), amyloid was present in 34/37 males versus 5/14 females in one line, and circulating human transthyretin concentrations were 30–50% lower in females across lines; cross-line comparisons suggested sex-related effects on deposition that were not entirely explained by transthyretin concentration alone [27]. These preclinical findings indicate sex-associated differences in transthyretin levels and deposition, but they do not identify specific causal endocrine pathways in humans [12]. Accordingly, menopausal status has been discussed as a potential modifier of female cardiac involvement, but available evidence remains observational and does not prove a protective effect of estrogens on myocardial amyloid deposition [22].

Overall, the available literature supports associations between sex/hormonal status, circulating transthyretin levels, and ATTR-CA phenotype, while direct causal links between specific hormonal exposures and myocardial amyloid deposition in humans remain unproven [12]. Potential mechanisms discussed in the literature include hormone-related modulation of circulating transthyretin availability and non–amyloid-specific effects on myocardial remodeling [23,24]. Sex-associated differences have also been described at the tissue level: proteomic profiling of human cardiac amyloid plaques identified differences between males and females in several non-TTR plaque proteins; the clinical implications of these differences are not yet established [28]. More broadly, sex differences in proteostatic stress responses have been described in other settings; however, ATTR-specific evidence linking these pathways to sex differences in cardiac transthyretin amyloid deposition remains limited [29].

**Figure 1:**
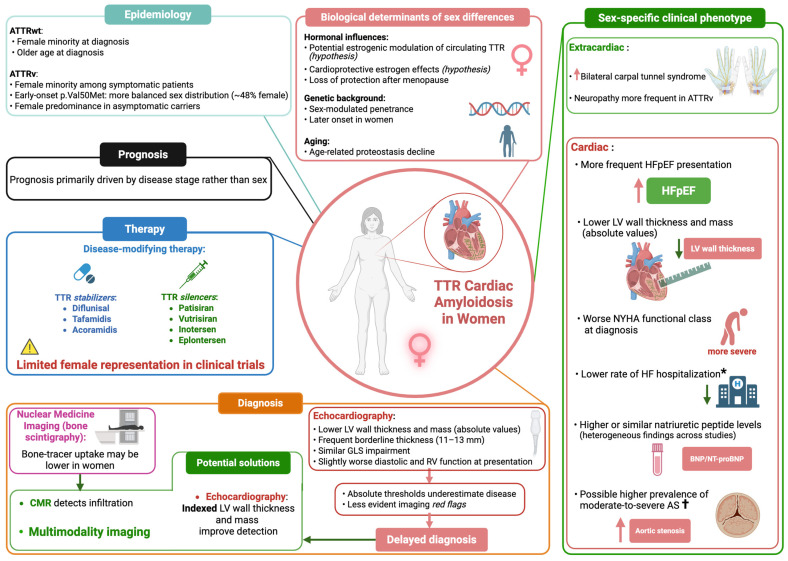
**(Central Illustration). Transthyretin Cardiac Amyloidosis in Women.** The figure summarizes the main clinical manifestations, phenotypic expression, diagnostic features, and therapeutic aspects of transthyretin cardiac amyloidosis in women. Comparisons refer to female versus male patients with transthyretin amyloidosis. Upward arrows (↑) indicate increased values, whereas downward arrows (↓) indicate reduced values. * Exploratory finding from the AMIGAL registry; to be interpreted with caution [30]. † Exploratory finding from the retrospective study by Takashio et al.; this sex-specific association remains uncertain and should be interpreted with caution [15]. AS, aortic stenosis; ATTRv, Hereditary transthyretin amyloidosis; ATTRwt, Wild-type Transthyretin Amyloidosis; BNP, B-type natriuretic peptide; CMR, Cardiac Magnetic Resonance; GLS, Global Longitudinal Strain; HF, Heart Failure; HFpEF, Heart Failure with preserved Ejection Fraction; LV, Left Ventricle; NYHA, New York Heart Association; NT-proBNP, N-terminal pro-B-type natriuretic peptide; RV, Right Ventricle; TTR, Transthyretin; p.Val50Met mutation in the TTR gene. This figure was created in BioRender. Barocelli, F. (2026). https://BioRender.com/p5glh9f.

Figure 1 (Central Illustration) summarizes phenotypic expression, and diagnostic features of TTR cardiac amyloidosis in women, highlighting key sex-related differences.

### 3.2. Genetic Background and Variant Distribution

The genetic penetrance and clinical expression of TTR variants show significant and clinically relevant differences between the sexes, with important implications for genetic counseling, family screening, and clinical management.

ATTR amyloidosis is caused by variants in the TTR gene located on chromosome 18, which comprises four exons and produces TTR monomers that assemble into homotetramers [31,32]. The disease is inherited in an autosomal dominant manner but is characterized by incomplete penetrance and variable expressivity [31,33]. Over 130–150 pathogenic variants of the TTR gene have been identified, most of which promote protein misfolding and result in ATTR amyloidosis. Genetic variants represent single-nucleotide changes in the TTR gene that result in amino acid substitutions, destabilization of TTR, and protein misfolding [32]. Most TTR variants are associated with ATTRv polyneuropathy, ATTRv cardiomyopathy, or mixed ATTRv systemic amyloidosis, but some patients with TTR mutations present with ocular amyloidosis or central nervous system involvement [34].

In autosomal dominant ATTRv amyloidosis, sex is not expected to determine the prevalence of pathogenic TTR variant carriage. Rather, sex-related differences mainly concern disease penetrance, cardiac phenotypic expression, and severity of myocardial involvement. In the THAOS registry, which represents the largest systematic analysis of the role of sex in ATTRv, the overall male-to-female distribution was essentially balanced when both patients with ATTRv and asymptomatic carriers were considered (53.6% male vs. 46.4% female) [21]. However, male representation increased among patients with symptomatic ATTRv (59.0% men vs. 41.0% women) and was even higher in patients with mixed phenotypes (63.5%) and predominantly cardiac phenotypes (68.7%). These findings suggest that sex-related differences in ATTRv mainly reflect differences in clinical penetrance and cardiac phenotypic expression rather than an intrinsically higher prevalence of pathogenic TTR variant carriage in men [21]. Notably, the proportion of men reached 72.2% among patients with ATTRv and cardiomyopathy defined as left ventricular wall thickness (LVWT) > 12 mm. This suggests that the relationship between sex, genetics, and disease expression is not entirely mediated by the prevalence of male patients with certain mutations, but also involves genetic factors not limited to TTR as well as non-genetic factors [21].

The distribution of TTR variants shows sex-specific patterns. The p.Val50Met variant, the most common worldwide, was present in both sexes with a male-to-female ratio of 0.97, although its frequency was lower in men (60.2%) than in women (72.0%) [21]. This variant, originally described in Portugal, affects approximately 1 in 500 individuals in northern Portugal, and approximately 1 in 1 million people in Japan [32]. In the study by Olsson et al. (2014) [35], 3460 samples from the Northern Sweden Health and Disease Study cohort (a population from northern Sweden) were genotyped. An overall carrier frequency of 1.82% for the p.Val50Met variant was observed. This figure is remarkably high and further underscores the highly variable penetrance of the disease.

The penetrance of p.Val50Met varies considerably by variant and geography. In Portugal, the penetrance of early-onset p.Val50Met is estimated at 80% by age 50 and 91% by age 80, whereas in Sweden, penetrance is much lower: 1.7% by age 30, 5% by age 40, 11% by age 50, 22% at age 60, 36% at age 70, 52% at age 80, and 69% at age 90 [31,34]. Anticipation (earlier onset of the disease in subsequent generations) is well documented for p.Val50Met and is more prominent in men with maternal transmission. The male prevalence was higher in non p.Val50Met cardiac mutations (p.Val142Ile, p.Leu131Met, p.Thr80Ala(Thr60Ala), or p.Ile88Leu) and in the p.Phe84Leu (Phe64Leu) and p.Ile127Val (Ile107Val) mutations [31]. When considering only patients with symptomatic ATTRv, the male prevalence in these mutations was typically even higher, with a male-to-female ratio of 6.50 in patients with p.Ile127Val, 3.42 for p.Val142Ile, 3.30 for p.Ile88Leu, 2.29 for p.Phe84Leu, 1.80 for p.Leu131Met, and 1.69 for p.Thr80Ala [21].

Among patients with ATTRv in the genotypic subgroups, the proportion of men increased progressively, from 50.6% in patients with early-onset p.Val50Met (the most “neurogenic” mutation) to 61.2% in non p.Val50Met non-cardiac cases, to 63.9% in late-onset p.Val50Met, and up to 73.2% among cardiac mutations [21]. The p.Val142Ile variant is the most common TTR variant in the United States, observed in approximately 3.4% of African Americans, corresponding to about 1.5 million people [32,36]. This variant, which originated on the west coast of Africa, is associated with the development of ATTR cardiomyopathy later in life, typically after age 60 [32].

There is considerable variation in the reported penetrance of p.Val142Ile, ranging from 7% (based on parietal thickening demonstrated by echocardiography) to 39% (based on nuclear scintigraphy) up to 100% (autopsy samples) [32]. Selected autopsy data suggest that older carriers of the p.Val142Ile variant, historically referred to as Val122Ile, may frequently show subclinical cardiac amyloid deposition, although this observation should not be generalized to all carriers [37].

Age and sex play an important role in the penetrance of p.Val142Ile, with male carriers being more severely affected. In a large national cohort, it was found that older Black women who were carriers of the p.Val142Ile variant had a substantially higher risk of cardiovascular disease and mortality compared to non-carriers. However, earlier studies such as ARIC, which included 124 individuals with a mean age of approximately 50–53 years (about 64% women), showed only a moderately elevated risk of heart failure (HF) (HR: 1.47) [37].

Recent data from the REGARDS study, which included 232 carriers (approximately 61% women) of similar age but with a shorter follow-up period, indicate a strong association with HF (HR: 2.43) and mortality (HR: 1.46), consistent with the most recent observations [37].

Despite intensive investigations, few clear genotype-phenotype correlations have been identified. Most pathogenic TTR variants result in peripheral and autonomic neuropathy, but some variants have been associated with phenotypes in which peripheral or autonomic neuropathy is clinically absent or less prominent. A predominantly cardiac phenotype is associated with specific variants, including p.Asp38Asn, p.Val40Ile, p.Pro44Ser, p.Ala65Ser, p.Ala65Thr, p.His76Arg, p.Gly77Arg, p.Ile88Leu, p.Ala101Thr, p.Ala101Val, p.His108Arg, p.Glu112Lys, p.Arg123Ser, p.Leu131Met, or p.Val142Ile. In individuals with these variants, peripheral and autonomic neuropathy is absent or less evident [34].

The composition of amyloid fibrils adds another layer of complexity. In Swedish patients with Val50Met, two types of fibrils have been identified: type A, consisting of a mixture of truncated and full-length ATTR fibrils, and type B, consisting of full-length fibrils. A study of 107 Swedish patients with ATTR p.Val50Met showed that there was no significant difference in the proportions of the two types of fibrils between men and women [38]. Nonetheless, in patients with type A fibrils, women had significantly lower median septal and posterior wall thicknesses (*p* = 0.007 and *p* = 0.010), lower height-indexed left ventricular mass (*p* = 0.008), and higher septal strain (*p* = 0.037) compared to men. These differences were not evident in patients with type B fibrils. Multiple linear regression analysis revealed that fibril type, sex, and age all had a significant impact on left ventricular septal thickness [38]. This study [38] demonstrates a clear gender difference in the severity of amyloid cardiac disease in patients with ATTR p.Val50Met amyloidosis. Although type A fibrils were associated with more advanced amyloid cardiac disease than type B fibrils, women with type A fibrils generally developed less cardiac infiltration than men.

### 3.3. Aging and Comorbidities

Aging and environmental factors are critical determinants in ATTR-CA, with significant sex differences that influence both the pathogenesis and clinical recognition of the disease.

Aging is the primary risk factor for the development of ATTR-CA, through complex biological mechanisms that facilitate the formation and deposition of amyloid fibrils. The dissociation of the TTR tetramer into its constituent subunits represents the rate-limiting step in amyloid fibril formation, while proteolytic cleavage of TTR further promotes this process [32].

With advancing age, impaired proteostatic homeostasis, including endoplasmic reticulum-associated degradation of misfolded TTR, progressively contributes to the development of amyloidosis [32]. The underlying mechanisms leading to the development of cardiac ATTRwt are still a matter of debate, but aging may destabilize TTR through post-translational biochemical alterations in the protein itself or in its chaperones [39].

ATTR-CA develops over the course of years or even decades, often evading clinical diagnosis. TTR deposits accumulate among cardiac myocytes in the interstitial space, contributing to diastolic dysfunction, progressive ventricular wall thickening, and impaired longitudinal systolic function. A clinically relevant finding is that amyloid fibril deposition in ligamentous structures often precedes myocardial deposition by approximately 5–15 years, offering a time window for early identification of the disease through extracardiac manifestations such as CTS [32].

Women with ATTR-CA are consistently diagnosed later than men, with an average difference of 3–3.3 years. For both wild-type and variant forms, the mean age at diagnosis ranges from 74 to 90 years, but women tend to present approximately 3 years later than men: the mean age is 82 vs. 78 years for ATTRwt and 77 vs. 75 years for ATTRv with the p.Val142Ile genotype [9,32].

In a study by the UK National Amyloidosis Centre [9] involving 1732 consecutive patients, women were 3.3 years older than men at presentation, with consistent differences across the various genotypes: ATTRwt-CA (81.9 vs. 77.8 years), p.Thr80Ala ATTR-CA (68.7 vs. 65.1 years), and p.Val142Ile ATTR-CA (77.1 vs. 74.9 years). This age difference may reflect either slower disease progression in women, potentially mediated by premenopausal hormonal protection, or a diagnostic delay due to atypical presentations, gender-unadjusted diagnostic thresholds, or underestimation of symptoms in older female patients.

Moreover, findings from autopsy studies provide further valuable evidence regarding the actual prevalence of myocardial TTR fibril deposition, reinforcing the notion that the condition is substantially underdiagnosed during life.

Approximately 25% of hearts from individuals who died after age 80 without known HF contained ATTRwt amyloid deposits, with a higher prevalence in older men (>70 years) with HF [40]. In other autopsy series, the incidence of myocardial ATTRwt deposits increased with age, with a prevalence of up to 20–25% in octogenarians and 37% in those aged > 95 years. Considering the association between cardiac amyloid deposition and HFpEF, a Mayo Clinic’s post-mortem autopsy study [13] has been conducted in patients with a prior diagnosis of HFpEF without clinically evident amyloidosis. 109 patients and 20 control subjects without an ante-mortem diagnosis of HF were included as age- and sex-matched controls and amyloid type and fibrosis severity were determined. Overall, similar rates of wtTTR amyloid deposition in the LV were observed in both sexes (19% in men and 15% in women). Among patients > 80 years of age, the incidence of ATTRwt deposits increased to 40%, with a marked prevalence in males. Specifically, moderate-to-severe interstitial TTR amyloid deposition, which would be consistent with a diagnosis of wilde-type cardiac amyloidosis, was the least frequent finding (5%) and showed a marked male predominance (80%). Mild interstitial deposits and/or variable degrees of wtTTR deposition within the intramural coronary vessels were more common in patients with HFpEF than in control subjects. Furthermore, the presence of wtTTR amyloid, although often mild, was associated with greater myocardial fibrosis and lower cardiac weight than expected after adjustment for age, sex, and body size [13].

The autoptic study of Cianci et al. [41], involving patients aged ≥ 80 years, did not demonstrate significant sex-related differences in the rates of myocardial amyloid detection. However, amyloid infiltration was more severe in men than in women, with extensive deposition observed in 10% of affected males compared with only 1% of females. This finding was associated with greater disease severity in male patients.

Finally, it is worth mentioning the autopsy series reported by Roberts et al. in 1998 [42], which evaluated 490 unselected elderly individuals aged over 80 years. Cardiac amyloidosis was identified both macroscopically and histologically in the myocardium of 10% of patients who died from cardiac causes. Furthermore, histological examination of many patients within the cohort revealed the presence of small aggregates of amyloid fibrils that were not associated with any overt clinical manifestations.

However, clinical cohorts have shown inconsistent results regarding whether ATTRwt cardiomyopathy is more common in males. In two case series of 229 patients with ATTRwt cardiomyopathy referred to specialized treatment centers: 81–98% of the patients were male [32]. This discrepancy between autopsy and clinical data suggests that women with ATTR-CA may be systematically underdiagnosed during their lifetime, likely due to diagnostic criteria that are not optimized for the female population. Environmental factors and comorbidities may affect the sexes differently and contribute to underdiagnosis in women. ATTR-CA often develops in patients with other common conditions, such as hypertension, which can cause left ventricular wall thickening and prevent the identification of ATTR-CA.

Recent studies have documented a significant increase in the rate of ATTR-CA diagnosis among women, suggesting that previously reported prevalence figures may have underestimated the true incidence of the disease in women.

An observational study by Prasad et al. [43] of 140 consecutive patients with ATTR-CA diagnosed between 2005 and 2022 at the Oregon Health and Science University Amyloidosis Clinic showed an increase in the proportion of women diagnosed from the pre-2019 period to 2019–2022: from 5.9% (4/68) to 16.7% (12/72) in the overall cohort and from 0% (0/51) to 11.3% (7/62) in the wild-type subgroup. 2019 was the year of tafamidis’s approval by the FDA, suggesting that increased awareness and the availability of effective therapies contributed to the rise in recognition [43].

## 4. Sex-Specific Clinical Phenotype of ATTR-CA

### 4.1. Heart Failure Presentation (HFpEF vs. HFrEF)

Women with ATTR-CA exhibit distinctive features in the presentation of HF, with important implications for diagnosis and clinical management.

The primary manifestation of ATTR-CA is HF, typically HFpEF in the early stages of the disease, accompanied by conduction abnormalities such as heart block and arrhythmias such as atrial fibrillation or ventricular tachycardia. Women generally present with a history of HFpEF and/or CTS [12]. Patients with ATTR-CA commonly present with dyspnea, fatigue, and edema, but these findings are nonspecific and are often misdiagnosed as non-amyloid HFpEF, representing a missed opportunity [44]. The study by González-López et al. [45] on 108 consecutive patients with ATTRwt demonstrated that the clinical spectrum of the disease is significantly more heterogeneous than traditionally believed. Contrary to the classic phenotype described in the literature: 19% of patients were women (20 out of 108), a significantly higher proportion than previous estimates of 5–10%; 23% exhibited a pattern of asymmetric hypertrophy, challenging the notion that concentric hypertrophy is a universal feature; 37% had an LVEF < 50%, indicating that reduced ejection fraction is not uncommon; only 20% met the criteria for low-voltage QRS, while 10% showed left ventricular hypertrophy on ECG; 32% had previously been misdiagnosed, highlighting the diagnostic challenges [45]. These data demonstrate that ATTRwt is not exclusively a disease of elderly men with concentric hypertrophy, preserved LVEF, and low QRS voltages, but presents a much broader clinical spectrum that includes a significant proportion of women [45].

Women with ATTR-CA have significantly higher left ventricular ejection fractions (LVEF) than men in multiple studies.

In the SCAN-MP study [44], women in the active surveillance cohort had an LVEF of 61% versus 50% in the reference cohort (*p* = 0.011). It is worth noting that women have smaller left ventricular cavities, smaller left ventricular end-diastolic diameters, and better-preserved ejection fractions compared to men.

A Japanese study by Yoshimura et al. [46] involving 106 consecutive patients with ATTRwt (including 12 women, 11.3%) confirmed that LVEF measured by CMR was significantly higher in women (59.5% [IQR 44.3–72.3%] vs. 50.0% [42.0–61.0%] in men, *p* = 0.04). Furthermore, the proportion of female patients increased with age: from 7.5% (6/80) in patients < 80 years to 23.1% (6/26) in patients ≥ 80 years [46].

Recent studies have revealed significant variability in HF phenotypes in ATTR-CA, challenging the traditional view of the disease as exclusively HFpEF. Relying solely on HFpEF for screening may lead to underdiagnosis, particularly in women.

### 4.2. Extracardiac Features in Women

Extracardiac manifestations are frequently observed in ATTR cardiomyopathy and may precede the cardiac diagnosis by several years, offering an opportunity for earlier disease identification [40].

Carpal tunnel syndrome (CTS) is a recognized extracardiac manifestation of ATTR-CA; among referred patients with ATTRwt-CA, CTS is reported in approximately 50% and typically precedes overt cardiomyopathy by an average of 5–10 years. In a German study [47] of 75 patients with ATTRwt, 84% had CTS (62% bilateral), with a median interval of 10 years between CTS surgery and the diagnosis of amyloidosis. Milandri et al. (*n* = 538) [48] compared the age- and sex-adjusted prevalence of CTS requiring decompression surgery across amyloidosis subtypes versus a published general-population cohort (14.9 million individuals) and found the highest prevalence in ATTR patients with cardiac involvement (20.3% vs. 4.1%). In ATTR, CTS standardized incidence ratios were markedly elevated in males in the eighth decade (13.08 in ATTRv and 15.5 in ATTRwt) [48]. Accordingly, although CTS is common in the general population (particularly in women), Milandri et al. showed that the excess prevalence of CTS in ATTR compared with the general population is mainly driven by older men, supporting higher diagnostic specificity of CTS as an ATTR-CM red flag in that subgroup. In a nationwide Danish cohort [49] of 56,032 individuals undergoing CTS surgery, CTS was associated with a higher subsequent risk of amyloidosis (HR 12.12), and there was no significant interaction with sex for the risk of amyloidosis and heart failure following CTS (interaction *p* = 0.5). These data support CTS surgery as an epidemiologic warning sign for future amyloidosis and heart failure in both women and men. A recent review [50] noted that bilateral CTS—particularly in men and when not explained by typical risk factors or when recurrent after surgery—should raise suspicion for amyloidosis; in women, this clue may be less readily recognized because idiopathic CTS is common. In the EDUCATE multicenter study [51] of unselected CTS decompressions (*n* = 555), amyloid was identified in 39% of biopsies, with higher positivity in men than women (51% vs. 30%), supporting higher specificity of CTS for amyloid in older men.

Regarding neurological manifestations, the THAOS study by Caponetti et al. [21] on patients with ATTRv demonstrated significant sex differences: a predominantly cardiac phenotype was more common in men (18.0% vs. 13.0%, *p* = 0.001), while a predominantly neurological phenotype was more common in women (64.6% vs. 57.4%, *p* < 0.001). This suggests that women with ATTRv may be more prone to neurological manifestations than cardiac ones. In ATTRwt, polyneuropathy is present in approximately 30% of patients, although it may be difficult to definitively attribute it to amyloidosis given the patients’ advanced age and frequent comorbidities [33]. A recent study [52] of 50 patients with ATTRwt (46 men, 4 women) found peripheral sensory polyneuropathy in 74% of cases, with mild-to-moderate severity and no significant motor involvement, and a low prevalence of autonomic symptoms compared to ATTRv. An interesting finding emerges from a Japanese study [53] on the progression of cerebral amyloid angiopathy in ATTRv: the annual rate of CAA progression was significantly higher in women than in men, suggesting that female sex may be associated with more rapid progression of central nervous system involvement [53].

Bilateral CTS, lumbar spinal stenosis, and spontaneous biceps tendon rupture are recognized extracardiac ‘red flags’ that should prompt consideration of cardiac amyloidosis (including ATTR-CM) in older patients with heart failure [54]. Besides, because CTS is common in women in the general population, its diagnostic specificity as a red flag for ATTR-CM may be lower in women, potentially contributing to under-recognition.

### 4.3. Functional Status and Disease Burden

Functional status and disease burden are crucial factors in the evaluation of patients with ATTR-CA, with significant sex-related differences emerging from recent literature.

One of the most relevant findings regarding sex-related differences in NYHA functional class comes from the Galician AMIGAL registry [30], which analyzed 385 patients with ATTR-CA (95 women, 290 men). Women were more frequently classified as NYHA class ≥ III compared to men (36.8% vs. 25.2%, *p* = 0.028), despite having a higher LVEF (56.0% vs. 52.6%, *p* = 0.003). This paradoxical finding suggests that women, despite having apparently better systolic function, exhibit greater functional impairment at diagnosis.

The THAOS study by Mora-Ayestaran et al. [55] confirmed this observation, demonstrating that the proportion of patients with NYHA Class III/IV increases progressively with age, reaching nearly 50% in older patients. Furthermore, the study highlighted a significantly longer diagnostic delay in younger women (approximately 5 years from symptom onset to diagnosis, compared to approximately 2 years in the other groups), suggesting that lower clinical suspicion in women contributes to a later diagnosis and thus a more advanced stage at presentation.

The study by Zampieri et al. [56] on 259 patients with ATTRwt reported that women, compared to men, were significantly older at diagnosis and had a higher NAC (National Amyloidosis Centre) score, suggesting more advanced disease at the time of recognition. However, biohumoral parameters, NYHA class, and ECG characteristics were similar between the sexes in ATTRv and AL amyloidosis.

Natriuretic peptides (BNP and NT-proBNP) are key biomarkers for assessing disease severity and prognostic stratification in ATTR-CA. BNP is elevated in 76% of patients with ATTR-CA and correlates significantly with septal thickness and basal septal strain, making it a sensitive marker for amyloid cardiomyopathy [57]. NT-proBNP correlates with left ventricular mass (calculated by CMR) and with late gadolinium enhancement, suggesting its utility as a measure of the severity of ATTR-CA. In a study of 36 carriers of mutant TTR [57] (asymptomatic or with neurological symptoms only), NT-proBNP showed a sensitivity of 92% and a specificity of 90% for predicting left ventricular echocardiographic abnormalities, allowing for the identification of the optimal time to initiate echocardiographic evaluation in carriers without cardiac symptoms. The NAC staging system uses NT-proBNP and eGFR to stratify patients into three prognostic stages: Stage I (NT-proBNP ≤ 3000 ng/L and eGFR ≥ 45 mL/min), Stage II (intermediate criteria), and Stage III (NT-proBNP > 3000 ng/L and eGFR < 45 mL/min) Median survival is 69.2 months for Stage I, 46.7 months for Stage II, and 24.1 months for Stage III (*p* < 0.0001). This system is applicable to both ATTRwt and ATTRv and represents a simple and universally available tool for prognostic stratification [58]. Recently, the NAC system has been expanded with the introduction of a Stage IV (defined as NT-proBNP ≥ 10,000 ng/L regardless of eGFR) to identify patients at high risk of early mortality [52]. NT-proBNP progression (defined as an absolute increase >700 ng/L and a relative increase >30% at 1 year) occurs in approximately one-third of patients and is associated with a 1.8-fold higher risk of mortality (HR 1.82; 95% CI 1.57–2.10; *p* < 0.001). NT-proBNP progression confers the highest risk in patients with Stage I (HR 2.34) and Stage II (HR 1.52) NAC disease, suggesting that this biomarker is particularly useful for identifying disease progression in patients with less advanced cardiac involvement [59]. Data on sex-related differences in BNP and NT-proBNP in ATTR-CA remain limited and not fully consistent. In the available cohorts discussed above, BNP was reported to be higher in women in one ATTRwt cohort, whereas THAOS data did not show significant sex-related differences in NT-proBNP levels in either ATTRwt or ATTRv [7,15]. Moreover, THAOS data [21] in ATTRv suggest that higher NT-proBNP quartiles are associated with greater male representation, probably reflecting more frequent cardiac phenotypic expression in men rather than a direct sex-specific difference in biomarker regulation. These findings should be interpreted cautiously because natriuretic peptide concentrations are influenced by age, renal function, atrial fibrillation, body size, and hemodynamic loading conditions.

In the AMIGAL registry [30], women with ATTR-CA had a poorer functional class at presentation but a lower incidence of HF hospitalizations than men (IR 167.39 vs. 245.61, *p* = 0.033), despite similar median survival (4.1 years in both sexes). This finding should be interpreted cautiously, as the available data do not allow conclusions regarding potential differences in treatment adherence, symptom reporting, care pathways, or hospitalization thresholds between women and men.

These data highlight the importance of careful assessment of functional status and biomarkers in both sexes. Women with ATTR-CA tend to present with a more advanced NYHA class despite an apparently better LVEF, a pattern that may be influenced by diagnostic delay, HFpEF-dominant presentation, and limitations of conventional systolic function parameters. The use of biomarker-based staging systems (NAC, Mayo) and monitoring of NT-proBNP progression are essential tools for prognostic stratification, although further studies are needed to determine whether sex-aware interpretation adjusted for major confounders can improve clinical assessment.

## 5. Sex Differences in Cardiac Imaging Findings

### 5.1. Echocardiographic Remodeling Patterns

Echocardiography is the first-line imaging modality for the evaluation of ATTR-CA, allowing for the assessment of cardiac structure and systolic and diastolic function [32]. ATTR-CA is characterized by biventricular wall thickening and myocardial stiffness, resulting in impaired relaxation. However, significant differences between the sexes emerge in echocardiographic remodeling patterns.

The seminal study by González-López et al. [45] debunked several myths about the disease, demonstrating that the clinical spectrum is more heterogeneous than previously believed. In this cohort, 19% of patients were female, a significantly higher proportion than previous estimates. Furthermore, a pattern of asymmetric hypertrophy was observed in 23% of patients; the mean LVEF was 52 ± 14%, with 37% of patients showing an LVEF < 50%, and only 20% met the criteria for low QRS voltage.

A study by the UK National Amyloidosis Centre [9] involving 1732 patients provided key data on sex differences in echocardiographic patterns. Women had lower absolute values for wall thickness than men, but when the parameters were indexed for body surface area, the overall structural and functional phenotype was similar between the sexes, with some significant differences suggesting a slightly worse phenotype in women. This study highlighted how unindexed measurements of wall thickness may have contributed to both underrepresentation and diagnostic delays in women.

The study by Zampieri et al. [56] involving 259 patients with ATTRwt confirmed that women, compared to men, exhibited a thicker normalized interventricular septum (IVS), more pronounced diastolic dysfunction, and poorer right ventricular function. These data suggest that women are diagnosed at a more advanced stage of the disease.

The review by Aimo et al. [14] summarized sex differences in echocardiographic patterns: women generally have smaller left ventricular cavities, better-preserved ejection fractions, and apparently slightly poorer right ventricular and diastolic function. When indexed, left ventricular wall thickness is equal to or even greater than that in men.

The SCAN-MP study [44] compared an active case-finding cohort with a clinical referral cohort, demonstrating that women with ATTR-CA in the active case-finding cohort had a higher LVEF (61% vs. 50%, *p* = 0.011), a lower left ventricular mass index (LVMi) (110 vs. 148 g/m^2^, *p* = 0.014), and a thinner posterior wall (1.4 vs. 1.6 cm, *p* = 0.01) compared to women in the reference cohort. This suggests that active screening strategies can identify women at earlier stages of the disease.

A recent contribution comes from the study by Papatheodorou et al. [60] which systematically evaluated 95 patients with confirmed ATTR-CA (69 men, 26 women, mean age 81.8 ± 7.6 years), all with Perugini grade 3. This study revealed that male patients had significantly higher rates of reduced LVEF (<50%) compared to women (36.2% vs. 15.4%, *p* = 0.04). This finding confirms that women tend to maintain better-preserved systolic function.

### 5.2. Indexed vs. Absolute Wall Thickness

One of the most critical issues in the diagnosis of ATTR-CA in women concerns the use of absolute vs. indexed values for wall thickness.

The 2023 ACC guidelines recommend considering an increased left ventricular wall thickness “above the sex-specific upper normal limit and typically ≥1.2 cm” as a diagnostic clue [33]. However, since normal wall thickness values are lower in women, applying the same cutoffs for both sexes could lead to underdiagnosis or delayed diagnosis in women [14]. In the ASE/EACVI chamber quantification recommendations, normal IVSd is lower in women than in men (0.6–0.9 cm vs. 0.6–1.0 cm) [55]. Furthermore, prior studies have reported sex- and age-specific reference values for IVSd indexed to BSA (mm/m^2^): 4.2 (0.6) in women < 40 years and 4.5 (0.7) in women 40–60 years, compared with 4.3 (0.6) and 4.6 (0.7) mm/m^2^ in the corresponding male age groups [56]. These normative differences emphasize that identical absolute wall thickness values may represent a larger deviation from normal in women, supporting the clinical rationale for indexing septal thickness by BSA when interpreting suspected ATTR-CA.

The study by Aimo et al. [14] on 330 consecutive patients with ATTR-CA specifically investigated this issue. IVS and posterior wall (PW) thickness values were lower in women (*n* = 53, 16%) than in men, but most of the differences were eliminated when the values were indexed for body surface area (BSA), height, or height^2^. Significantly, PW thickness indexed by height^2^ was actually higher in women, suggesting similar disease severity when accounting for smaller body size. Moreover, IVS values normalized by height^2^ showed stronger associations with NT-proBNP levels than unnormalized values. Similarly, indexed values showed stronger correlations with relative wall thickness (RWT), the E/e’ ratio, and TAPSE. These data strongly support the use of indexed echocardiographic parameters for a more accurate assessment of disease severity, particularly in women.

The recent study by Chedid El Helou et al. [61] involving 1845 patients confirmed that a significant proportion of patients present with normal or mildly increased wall thickness (≤1.2 cm), including 6.5% of patients with ATTR-CA. Women had significantly lower wall thickness. However, RWT was more sensitive than the LVMi in identifying ATTR-CA and showed no gender differences. These data suggest that RWT may be a more reliable parameter for screening in women.

### 5.3. Myocardial Strain and Right Ventricular Involvement

The pattern of longitudinal strain with “apical sparing” is one of the most characteristic echocardiographic features of ATTR-CA. Amyloid fibril deposition proceeds from the base to the apex and from the endocardium to the epicardium; since the longitudinal contractile fibers are located predominantly in the endocardium, longitudinal contraction is compromised early in the disease process, while radial contraction is preserved until the terminal stages [32].

The study by Yoshimura et al. [46] enrolling 106 patients with ATTRwt (12 women, 11.3%), specifically evaluated sex differences in imaging parameters. No significant sex differences in GLS were observed between men and women. However, women had a significantly higher LVEF on CMR (59.5% vs. 50.0%, *p* = 0.04).

Papatheodorou et al. [60] described a left ventricular apical sparing-like tracer uptake pattern on planar and SPECT imaging in 29% of patients with ATTR-CM, with no statistically significant sex-related difference. In the present review, this nuclear imaging finding is discussed separately from echocardiographic relative apical sparing assessed by longitudinal strain imaging.

The study by Ozbay et al. [62] demonstrated that right ventricular free-wall strain has incremental value compared to relative apical sparing of the left ventricle for the differential diagnosis of ATTR-CA among phenotypes of left ventricular hypertrophy. An RV free-wall strain ≥ −16% was predictive in addition to LV RAS in the overall group and in the subgroup without extreme wall thickness (≤14 mm).

Right ventricular involvement represents a crucial prognostic factor in ATTR-CA. The study by Porcari et al. [63] involving 1422 patients demonstrated that right ventricular uptake on bone scintigraphy was detectable in 100% of cases at diagnosis using SPECT imaging. Diffuse right ventricular uptake (*n* = 936) was associated with higher all-cause mortality compared to focal uptake (*n* = 486) (77.9% vs. 22.1%, *p* < 0.001). In multivariate analysis, diffuse right ventricular uptake remained an independent predictor of all-cause mortality (HR 1.60; 95% CI 1.26–2.04; *p* < 0.001). The study by Holcman et al. [64] confirmed that right ventricular uptake on SPECT/CT with 99mTc-DPD was present in 91% of patients with ATTR-CA. Right ventricular uptake was associated with increased right ventricular wall thickness, left ventricular GLS, and NT-proBNP levels. Multivariate analysis identified increased LVMi and NYHA class as predictors of right ventricular involvement.

The study by Papatheodorou et al. [60] also provided specific data on right ventricular uptake in relation to sex: right ventricular uptake was observed in 39% of the overall cohort, with no statistically significant differences between males and females. This finding suggests that right ventricular involvement, as assessed by nuclear imaging, does not exhibit a significant sex-specific prevalence.

About right ventricular function as assessed by echocardiography, the study by Zampieri et al. [56] reported that women with ATTRwt had poorer right ventricular function than men. This finding was confirmed by the review by Aimo et al. [12], which highlighted that women appear to have slightly poorer right ventricular and diastolic function. On the other hand, in the spanish AMIGAL registry [30], published in 2026, no difference in right ventricular function was observed between the sexes.

The discrepancy between scintigraphic uptake (similar between the sexes) and echocardiographic function (worse in women) may reflect the fact that women are diagnosed at more advanced stages of the disease.

### 5.4. Implications of Multimodality Imaging

CMR can directly visualize and quantify the expansion of the interstitial space caused by the deposition of amyloid fibrils between cardiomyocytes [32]. LGE in ATTR-CA is typically diffused, with subendocardial and transmural enhancement patterns that appear distinct from those of other myocardial processes. Measuring myocardial T1 time before and after contrast administration allows quantification of the extracellular volume fraction (ECV), which is typically >40% in ATTR-CA compared to the normal range of <30–32% [32].

The study by Yoshimura et al. [46] provided specific data on sex differences in CMR parameters. Women exhibited significantly higher LVEF (59.5% vs. 50.0%, *p* = 0.04), significantly lower ECV (50.4% vs. 54.2%, *p* = 0.04), less extensive LGE compared to men, no significant difference in native T1 and myocardial T2. These data suggest that women have a milder myocardial amyloid burden at the time of diagnosis, which may reflect earlier diagnosis or slower disease progression. However, these parameters could also reflect a later diagnosis resulting in the selection of less severe cases.

Scintigraphy using 99mTc-labeled bone tracers (PYP, DPD, HMDP) is a cornerstone of the noninvasive diagnosis of ATTR-CA. In the absence of light-chain abnormalities, a grade 2 or 3 cardiac uptake on bone-tracer scintigraphy is highly specific for ATTR-CM only when monoclonal gammopathy has been excluded and image acquisition and interpretation are appropriate, preferably including SPECT or SPECT/CT confirmation of true myocardial uptake [65,66].

The study by Yoshimura et al. [46] demonstrated that the heart-to-contralateral ratio in 99mTc-PYP scintigraphy was significantly lower among female patients (1.64 vs. 1.88, *p* = 0.02). This finding, together with lower ECV on CMR, indicates a milder myocardial amyloid burden in women.

The study by Papatheodorou et al. [60] provided relevant information on extracardiac soft tissue uptake: male patients showed significantly higher rates of soft tissue uptake compared to women (53.6% vs. 30.8%, *p* = 0.04). This finding is particularly interesting because extracardiac soft tissue uptake may reflect more extensive systemic involvement of the disease in men. This paper also highlighted significant sex differences in conduction abnormalities: male patients had significantly higher rates of atrioventricular block than women (27.5% vs. 0%, *p* = 0.003). This marked difference suggests that conduction abnormalities may be a marker of more advanced disease or a different pattern of amyloid deposition in men.

A multimodal imaging approach in ATTR-CA is essential for accurate diagnosis and optimal prognostic stratification. Indeed the 2021 ASNC/AHA/ASE/EANM/HFSA/ISA/SCMR/SNMMI guidelines recommend the integration of echocardiography, CMR, and bone scintigraphy for the comprehensive evaluation of patients with suspected ATTR-CA [65].

In conclusion, multimodal imaging reveals that women with ATTR-CA exhibit a distinct imaging phenotype characterized by higher LVEF, lower ECV, less extensive LGE, weaker scintigraphic uptake, and lower uptake in extracardiac soft tissues. The study by Papatheodorou et al. [60] also demonstrated that, although most ECG and imaging phenotypes are equally distributed between the sexes, certain specific markers, such as reduced LVEF, atrioventricular block, and soft tissue uptake, show a sex-specific prevalence with a male predominance. These data suggest that men tend to present with a more advanced disease phenotype at the time of diagnosis, while women may be underdiagnosed due to diagnostic criteria not optimized for the female population. The adoption of sex-specific criteria and the use of indexed parameters are essential for improving diagnosis in women.

## 6. Therapeutic Response

### 6.1. Available Evidence on Disease-Modifying Therapies

The therapeutic breakthrough in halting disease progression occurred with the approval of tafamidis, the first drug authorized by both FDA and EMA. This therapy has been validated in both forms of ATTR-CA (wild-type and variant-mutated). Tafamidis is a disease-modifying agent that acts as a TTR stabilizer by binding to the thyroxine-binding sites of the TTR tetramer, thereby preventing its dissociation, inhibiting subsequent amyloid fibril formation and cardiac deposition and slowing the progression of ATTR-related polyneuropathy [67]. The ATTR-ACT trial [68] demonstrated that, compared with placebo, tafamidis improved overall survival, reduced hospitalizations due to HF, enhanced quality of life in treated patients and slowed the decline in 6MWD. These benefits were observed with both the 80 mg and 20 mg doses, including attenuation of functional capacity decline. Notably, the 80 mg dose achieved improvement in quality of life earlier than the 20 mg dose (6 months vs. 12 months). Including the long-term extension study to evaluate outcomes with a longer duration of treatment, a significant reduction in the risk of death was observed with tafamidis 80 mg compared to 20 mg (30% relative reduction). This is due to the dose-dependent stabilization effect of the tetramer. Indeed, a higher mean concentration of tetrameric TTR was observed with the 80 mg dose. Confirming these observations, a significant reduction in NT-proBNP and troponin I was observed with the higher tafamidis dose [67]. Currently, the 61 mg tablet formulation is also available. Regarding female patients, in ATTR-ACT trial there was poor representation of this population. In fact, only 43 women out of 441 patients were enrolled (9.7%). No statistically significant data by gender were reported. In the predictive model of Monteiro et al. [69], female gender, higher TTR concentrations and lower disease severity predict a positive response to tafamidis in ATTRv with polyneuropathy. On the other hand, such results were not seen in ATTR-CA. In the study by Vong et al. [70], predictors of survival were analyzed in patients with ATTR-CA treated with tafamidis. It was found that the wt genotype, better 6MWT results, higher LVEF, and lower BUN and NT-proBNP concentrations were associated with a lower risk of death whereas lower troponin I levels were associated with a lower risk of hospitalization. Conversely, no differences in therapeutic response were referred based on gender. Finally, in the multicenter international cohort study published by Debonnaire et al. [71], 1454 patients with ATTRwt-CM were included, of whom 307 (21.1%) were female. At presentation, female patients were, on average, 3 years older than men, had more advanced disease (including higher indexed left ventricular wall thickness), and more frequently exhibited HFpEF and arterial hypertension. The natural course of the disease was poor, with no significant sex-related differences. Tafamidis was initiated in 1055 of the 1454 patients and was prescribed 12% less frequently in female patients than in male patients (*p* < 0.001). As no significant sex-related differences were observed in the reasons for treatment non-initiation (*p* = 0.116) or discontinuation (*p* = 0.304), the authors interpreted these findings as potentially reflecting persistent undertreatment of female patients. Furthermore, no statistically significant treatment-by-sex interaction was observed (*p* = 0.381), and female sex was not an independent predictor of mortality among treated patients (*p* = 0.365).

Acoramidis is another TTR stabilizer currently indicated for both wt and variant ATTR-CM. Compared with tafamidis, it binds to the protein with greater selectivity, mimicking the protective T119M variant, which can prevent amyloid deposition [72]. In the Phase III ATTRibute-CM clinical trial [73], acoramidis achieved significant results compared to placebo in reducing all-cause mortality, cardiovascular hospitalizations, NT-proBNP, and improving 6MWT distance. Even in this trial the female population was poorly represented (9.8%, 62 patients, total 632) and no statistically significant sex-by-treatment interaction was reported.

On the other hand, gene-silencing therapies do not prevent TTR dissociation and subsequent amyloid formation; rather, they directly reduce hepatic production of the protein.

Patisiran is a small interfering RNA (siRNA) administered intravenously every 3 weeks currently approved for the treatment of ATTRv polyneuropathy [74]. It is delivered via nanoparticles and acts via the RNA-induced silencing complex (RISC) to degrade TTR mRNA [74]. In the APOLLO trial [75], patisiran demonstrated the ability to reduce serum TTR levels by up to 80%, improving neuropathy and decreasing NT-proBNP levels in patients with cardiac involvement. However, patisiran has shown frequent infusion-related adverse reactions, which required vitamin A supplementation and premedication. In this main trial female population accounted for 26% (58, total 225). No differences between males and females in terms of response to patisiran were related. Subsequently, in 2023, the APOLLO-B trial [76], a multicenter, randomized phase 3 and double-blind study, was published. The trial enrolled patients with ATTRv and ATTRwt-CA. At 12 months, in the patisiran-treated group, a mean percentage reduction of 86.8% in serum ATTR levels was observed. Moreover, treatment with patisiran slowed disease progression compared with placebo and improved patients’ functional capacity and quality of life. A total of 359 patients were enrolled in APOLLO-B. Of these, 38 were female (10.6%), and no differences in outcomes were reported in this subgroup. As with the other major trials, sex-specific analyses were limited and underpowered.

Vutrisiran is a second-generation siRNA. It targets the liver through conjugation with a triantennary N-acetylgalactosamine (GalNAc) ligand, which binds to the asialoglycoprotein receptor expressed on the surface of hepatocytes [74]. The siRNA–GalNAc conjugate incorporates an enhanced stabilization chemistry that increases its potency and confers high metabolic stability, allowing for subcutaneous administration once every three months. Vutrisiran is approved for the treatment of polyneuropathy in adults with ATTRv cardiomyopathy. In the multicenter, randomized phase 3 HELIOS-A trial [77], vutrisiran was compared with the external placebo group of the APOLLO trial in patients with documented ATTRv polyneuropathy (and with adequate hepatic/renal function, NYHA class ≤ 2). Vutrisiran demonstrated a rapid and sustained reduction in serum TTR levels. In parallel, a decrease in serum vitamin A levels was observed. The drug was associated with significant improvements in neuropathy, quality of life, gait, and disability compared with the untreated group; these benefits were evident as early as 9 months after treatment initiation. Moreover, compared to patisiran, vutrisiran does not require premedication, although vitamin A supplementation may be necessary [72,77]. The percentage of female patients in the study amounted to 35.4% (58 patients out of a total of 164), and, in this case as well, no statistically significant interaction by sex was reported. HELIOS-B trial [78], a subsequent randomized, multicentre, phase 3 study, was conducted with the purpose to focuses on cardiovascular outcomes. A total of 654 patients with ATTR-CA were enrolled and randomized to receive vutrisiran (either as monotherapy or in combination with tafamidis) or placebo. Of these, only 49 were female, corresponding to 7.5% of the total. This trial showed a reduction in all-cause mortality and in recurrent cardiovascular events (both when vutrisiran was administered as monotherapy and combined with tafamidis) [78]. Even in the Helios-B cohort, as the study was not designed and nor did it have a sufficient statistical sample to establish a sex-by-treatment interaction, no differences based on gender were recounted.

Another class capable of silencing TTR gene expression is antisense oligonucleotides (ASOs). They bind TTR mRNA and induce its degradation through the recruitment of RNase H [74].

The phase 3 NEURO-TTR trial [79] evaluated inotersen (the first approved ASOs ATTRv PLN) in patients with ATTRv polyneuropathy. In this study, inotersen reduced TTR levels by 84%, with associated improvements in neuropathy and quality of life. Treatment with inotersen may lead to important adverse drug reactions, particularly thrombocytopenia and glomerulonephritis. This possibility therefore requires monitoring of platelet count and renal function [79]. In this trial female representation accounted for 31% (54 patients, total 172) and no statistically significant sex differences in response to inotersen were related.

The phase 3 NEURO-TTRansform trial [80] evaluated eplontersen, a GalNAc-conjugated antisense oligonucleotide, in 168 patients with ATTRv polyneuropathy. Eplontersen reduced serum TTR levels by 82% and halted neuropathy progression (mNIS+7: +0.3 vs. +25.1 with placebo; *p* < 0.001), with a substantially improved safety profile compared with inotersen, eliminating the need for mandatory platelet and renal function monitoring. Female representation accounted for 31% (45 of 144 patients in the eplontersen group). A dedicated exploratory analysis by Waddington Cruz et al. [81] demonstrated that eplontersen led to consistent TTR reductions (>80%) and halted neuropathy progression in both sexes, with comparable improvements in quality of life and functional outcomes. Female patients presented with less severe disease at baseline (lower Norfolk QoL-DN scores, higher proportion with PND score I), possibly reflecting the potential protective effects of estrogen and the differential impact of sex hormones on TTR levels; however, the treatment benefit of eplontersen was robust and independent of sex, including in patients with concomitant cardiomyopathy.

The phase 3 CARDIO-TTRansform trial [82] is currently underway to evaluate the efficacy and safety profile of eplontersen, an ASO that is more potent and has fewer adverse effects than inotersen, in patients with ATTR-CM. Eplontersen is currently approved for ATTRv polyneuropathy.

It is also worth focusing on the emerging gene-editing therapies currently under investigation based on CRISPR-Cas9 technology. Nexiguran ziclumeran is designed to permanently modify the TTR gene in hepatocytes, thereby suppressing the production of both ATTRwt and ATTRv [72]. A preclinical study in animal models have demonstrated that a single dose of the drug reduced serum TTR levels by up to 96% [83]. Clinical trials are currently ongoing to evaluate its efficacy and safety in patients with ATTR cardiomyopathy. In particular, the phase 3 MAGNITUDE clinical trial (NCT06128629) is underway to test the efficacy and safety of a single administration of NTLA-2001 compared to placebo in ATTR-CA.

Finally, anti-TTR antibodies specifically target the removal of amyloid deposits that have already accumulated in tissues. Consequently, they may represent a significant therapeutic advancement with the potential to render disease-related damage at least partially reversible. These antibodies act by stimulating the immune system to recognize and clear amyloid fibrils [72]. In the recent phase II trial [84] coramitug, a humanized monoclonal antibody targeting misfolded TTR, was well tolerated and, at a dose of 60 mg/kg, achieved a statistically significant reduction in NT-proBNP levels. However, no statistically significant improvement in 6MWT performance was observed at 52 weeks. The phase III CLEOPATTRA clinical trial (NCT07207811) is currently underway to evaluate coramitug versus placebo in patients with ATTR-CM. Approximately 1280 patients are expected to be enrolled. The phase III DepleTTR-CM clinical trial (NCT06183931) is currently underway to evaluate the safety and efficacy of the novel agent coramitug. In the case series reported by Fontana et al. [85], three male patients aged 68, 82, and 76 years with hypertrophic phenotype ATTR-CM experienced spontaneous resolution of HF despite not having received any disease-modifying therapy. Notably, endomyocardial biopsy from patient 3 revealed an infiltrate of macrophages and multinucleated giant cells surrounding the amyloid deposits. High-titer polyclonal IgG antibodies directed against human ATTR amyloid were detected in all patients. These antibodies showed specific binding to ATTR amyloid deposits in both murine and human models. However, it is important to acknowledge that similar antibodies have not been identified in patient cohorts evaluated either before or after this study, highlighting the extremely small sample size and limiting the generalizability of these findings. Table 1 summarizes the main clinical trials of ATTR-specific therapies, highlighting the representation of female patients and the availability of sex-specific analyses.

### 6.2. Limitations of Current Data

Despite the remarkable technological advances and the available evidence, none of the cited studies and trials have analysed the different pharmacokinetic and pharmacodynamic characteristics in men and women. Furthermore, the underrepresentation of the female sex in clinical trials is evident. Therefore, clear evidence is currently lacking regarding possible differences in response to modern disease-modifying therapies.

In any case, based on the currently available clinical data, the clinical therapeutic approach to be adopted is the same for both sexes.

## 7. Disease Progression and Prognostic Implications

Available evidence on sex-related differences in disease progression and prognosis in ATTR-CA [10,86,87] is largely derived from observational cohorts and registry-based analyses, with limited prospective or randomized data. Across the largest available studies [7,21,55,88], once age and disease severity at diagnosis are taken into account, disease trajectories and survival appear broadly comparable between women and men [9].

Reported sex-related differences in clinical outcomes are inconsistent across studies and are likely influenced by heterogeneity in study design, diagnostic thresholds, and referral patterns [12,22]. Differences in clinical presentation at diagnosis, including later age and less overt structural involvement in women, further support the hypothesis that observed variations in disease course may reflect differences in case ascertainment rather than intrinsic biological factors.

Overall, current evidence does not support a clearly defined sex-specific pattern of disease progression in ATTR-CA. However, interpretation of these findings remains limited by the observational nature of available data and by the underrepresentation of women in many clinical cohorts, which may affect the reliability of sex-stratified analyses.

## 8. Clinical Implications and Future Perspectives

### 8.1. Strategies to Improve Diagnosis in Women

Structured diagnostic strategies applied to patients with HFpEF or unexplained LVH identify a measurable proportion of previously unrecognized ATTR-CA cases [89,90]. Multicenter studies and meta-analyses show that when HFpEF patients undergo predefined diagnostic escalation with confirmatory testing, ATTR-CA emerges in a non-negligible subset [89,90].

Because women with ATTR-CA more often present with HFpEF-dominant phenotypes and less overt hypertrophy [9,15], structured HFpEF-based case-finding may improve diagnostic recognition in women without altering established confirmatory criteria. This approach does not imply indiscriminate screening but the systematic integration of predefined triggers within existing clinical pathways.

Misdiagnosis and delayed recognition remain documented challenges, with ATTR-CA frequently attributed to hypertensive heart disease, hypertrophic cardiomyopathy, or nonspecific HFpEF phenotypes [91,92].

Observational data confirm substantial diagnostic delay and initial misclassification [93]. Systematic use of predefined triggers may therefore improve case ascertainment among women whose structural expression deviates from classical hypertrophic patterns [9,15].

Extracardiac manifestations—including CTS, lumbar spinal stenosis, and neurologic features—often precede overt cardiac involvement and may lead patients into non-cardiology pathways [94,95,96]. Multidisciplinary awareness of these associations can facilitate earlier diagnostic consideration, particularly in women whose structural remodeling may be less conspicuous [9,53].

### 8.2. Toward Sex-Aware Management Pathways

Longitudinal management of ATTR-CA relies on consistent structural assessment over time. Sex-related differences in cardiac remodeling and body size may influence interpretation when absolute measurements are used [97,98,99].

Incorporation of indexed parameters and sex-aware reporting into routine follow-up may enhance comparability of disease severity and progression between women and men without modifying diagnostic thresholds. Such methodological consistency strengthens longitudinal monitoring and registry-based analyses [9].

### 8.3. Research Priorities: Strengthening Data Reliability and Generalizability

Current evidence on sex-related differences in ATTR-CA derives largely from observational cohorts and registries [9,10], with limited prospective studies designed for sex-stratified outcomes. Variability in diagnostic pathways and phenotypic capture may therefore affect representation of female presentations in clinical datasets.

Future research would benefit from systematic sex-stratified reporting of baseline characteristics, imaging parameters, and longitudinal outcomes in both registries and interventional trials [100,101,102]. This does not imply differential treatment strategies but enables more accurate interpretation of disease trajectories and therapeutic effects across the phenotypic spectrum.

Prospective designs incorporating predefined sex-based subgroup analyses and standardized data collection may further improve internal consistency and external validity in ATTR-CA research. Ensuring adequate female representation in future studies is essential to generate reliable and generalizable evidence and to accurately define sex-specific disease patterns in ATTR-CA.

## 9. Conclusions

Sex-related differences in ATTR-CA reflect a complex interplay between biological factors and diagnostic pathways, contributing to the persistent under-recognition of the disease in women. Maintaining a high index of clinical suspicion remains essential to ensure consideration of ATTR-CA in the differential diagnosis of women presenting with HF, particularly in those with HFpEF, especially when clinical features are subtle or nonspecific. Despite these differences in presentation, current evidence has not consistently demonstrated clear sex-specific differences in disease progression or outcomes once ATTR-CA is diagnosed, although available data remain limited and largely observational. The persistent underrepresentation of women in clinical studies limits the reliability and generalizability of the current evidence; therefore, future research should prioritize sex-balanced cohorts and dedicated analyses to better define disease trajectories and optimize management strategies in both sexes.

## Figures and Tables

**Table 1:** ATTR-specific therapies in the main clinical trials and representation of female patients within study populations.

Mechanism of Action	Trial	Drug and Investigated Populations	N m (%)/f (%)	Results (Primary Endpoints)	Sex Differences in Treatment Response	Reference
TTR tetramer stabilizers	ATTR-ACT Phase 3 (NCT01994889)	Tafamidis in pts with ATTR-CA (ATTRwt and ATTRv)	441 m 398 (90.3) f 43 (9.7)	Reduction in all cause-mortality and cardiovascular- related hospitalization.	N/A	Maurer M.S. et al. [68]
	ATTRibute-CM Phase 3 (NCT03860935)	Acoramidis in pts with ATTR-CA (ATTRwt and ATTRv)	632 m 570 (90.2) f 62 (9.8)	Benefits in the incidence of death from any cause, cardiovascular-related hospitalization, NT-proBNP level and 6MWD were observed in acoramidis group.	N/A	Gillmore J.D. et al. [73]
TTR silencers	APOLLO Phase 3 (NCT01960348)	Patisiran in pts with ATTRv and polineuropathy	225 m 167 (74) f 58 (26)	The change from baseline in the mNIS+7 was significantly lower in patisiran group.	N/A	Adams D. et al. [75]
	APOLLO B Phase 3 (NCT03997383)	Patisiran in pts with ATTR-CA (ATTRwt and ATTRv)	359 m 321 (89.4) f 38 (10.6)	The decline from baseline in the 6MWD was significantly lower in the patisiran group.	N/A	Maurer M.S. et al. [76]
	HELIOS A Phase 3 (NCT03759379)	Vutrisiran in pts with ATTRv and polineuropathy	164 m 106 (64.6) f 58 (35.4)	Vutrisiran met the primary endpoint of change from baseline in mNIS+7 at 9 months, improving neuropathy and QOL	N/A	Adams D. et al. [77]
	HELIOS B Phase 3 (NCT04153149)	Vutrisiran in pts with ATTR-CA (ATTRwt and ATTRv)	654 m 605 (92.5) f 49 (7.5)	Vutrisiran led to a lower risk of death from any cause and recurrent cardiovascular events.	N/A	Fontana M. et al. [78]
	NEURO-TTR Phase 3 (NCT01737398)	Inotersen in pts with ATTRv and polineuropathy	172 m 118 (69) f 54 (31)	Both mNIS+7 and Norfolk QOL-DN score showed significant benefits with inotersen treatment.	N/A	Benson M.D. et al. [79]
	NEURO-TTRansform Phase 3 (NCT04136184)	Eplontersen in pts with ATTRv and polineuropathy	172 m 118 (69) f 54 (31)	Eplontersen significantly reduced serum TTR levels, reduced neuropathy impairment, and improved quality of life.	Effective regardless of sex *	Coelho T. et al. [80]
	CARDIO-TTRansform (ongoing) Phase 3 (NCT04136171)	Eplontersen in pts with ATTR-CA (ATTRwt and ATTRv)	1438 N/A/N/A	N/A	N/A	N/A
Gene-editing therapies	MAGNITUDE (ongoing) Phase 3 (NCT06672237)	Nexiguran- ZIclumeran in pts with ATTRv and polineuropathy	N/A	N/A	N/A	N/A

Female representation is reported as available from published data. These trials were neither designed nor powered to assess a treatment-by-sex interaction; no specific sex-stratified analyses were reported within the primary trial publications. * Based on an exploratory analysis by Waddington Cruz et al. [81] ATTR, Transthyretin amyloidosis; ATTR-CA, Transthyretin Cardiac Amyloidosis; ATTRv, Variant (Hereditary) Transthyretin Amyloidosis; ATTRwt, Wild-Type Transthyretin Amyloidosis; f, females; mNIS+7, modified neuropathy impairment score +7; N, number of patients; m, males; N/A, not available; Norfolk QoL-DN, Norfolk Quality of Life-Diabetic Neuropathy; NT-proBNP, N-terminal pro-Brain Natriuretic Peptide; pts, patients; QOL, quality of life; TTR, transthyretin; 6MWD, 6 min walk distance.

## Data Availability

No new data were created or analyzed in this study. Data sharing is not applicable to this article.

## Abbreviations

The following abbreviations are used in this manuscript:

AA	Serum amyloid A amyloidosis
AApoA1	Apolipoprotein A-I amyloidosis
AH	Immunoglobulin heavy chain amyloidosis
AL	Light chain amyloidosis
ASO	Antisense oligonucleotide
ATTR	Transthyretin amyloidosis
ATTR-CA	Transthyretin Cardiac Amyloidosis
ATTRv	Variant (Hereditary) Transthyretin Amyloidosis
ATTRwt	Wild-Type Transthyretin Amyloidosis
BNP	B-type Natriuretic Peptide
BSA	Body Surface Area
CMR	Cardiac Magnetic Resonance
CRISPR-Cas9	Clustered Regularly Interspaced Short Palindromic Repeats-associated Protein 9
CTS	Carpal Tunnel Syndrome
ECV	Extracellular Volume
EMA	European Medicines Agency
FAP	Familial Amyloid Polyneuropathy
FDA	Food and Drug Administration
GalNAc	N-acetylgalactosamine
GLS	Global Longitudinal Strain
HF	Heart Failure
HFpEF	Heart Failure with Preserved Ejection Fraction
HFrEF	Heart Failure with Reduced Ejection Fraction
IVS	Interventricular Septum
LGE	Late gadolinium enhancement
LVEF	Left Ventricular Ejection Fraction
LVH	Left Ventricular Hypertrophy
LVMi	Left Ventricular Mass Index
NAC	National Amyloidosis Centre
NT-proBNP	N-terminal pro–B-type Natriuretic Peptide
NYHA	New York Heart Association
PLN	Polyneuropathy
Pts	Patients
QOL	quality of life
RISC	RNA induced silencing complex
RNA	Ribonucleic acid
RNAi	RNA Interference
RWT	relative wall thickness
siRNA	Small Interfering RNA
SPECT	Single Photon Emission Computed Tomography
THAOS	Transthyretin Amyloidosis Outcomes Survey
TTR	Transthyretin
6MWD	6-min walk distance
99mTc-PYP	Technetium-99m pyrophosphate
